# Upregulation of the TCA Cycle and Oxidative Phosphorylation Enhances the Fitness of CD99 CAR-T Cells Under Dynamic Cultivation

**DOI:** 10.3390/ijms27020607

**Published:** 2026-01-07

**Authors:** Jiaxuan Zhao, Youyong Wang, Yixuan Wang, Ge Dong, Han Wu, Yeting Cui, Lixing Gu, Fenfang Zhao, Guanlin Zhao, Jinyu Kang, Qian Zhang, Nan Liu, Ning Wang, Xiao Sun, Yao Xu, Tongcun Zhang, Jiangzhou Shi

**Affiliations:** 1 College of Biotechnology, Tianjin University of Science and Technology, Tianjin 300457, China; jiaxuan.zhao@mail.tust.edu.cn (J.Z.); wangyx668@163.com (Y.W.); 2 Institute of Biology and Medicine, College of Life Sciences and Health, Wuhan University of Science and Technology, Wuhan 430065, China; youyongwang999@163.com (Y.W.); wkd19890723@wust.edu.cn (G.D.); wuhan519131@163.com (H.W.); yetingcui09@wust.edu.cn (Y.C.); gulixing@wust.edu.cn (L.G.); zffang2025@wust.edu.cn (F.Z.); guanlin66@wust.edu.cn (G.Z.); kangjinyu@wust.edu.cn (J.K.); zq977977@wust.edu.cn (Q.Z.); liu_nanabc@126.com (N.L.); hhhh202511@163.com (N.W.); sunxiao940713@163.com (X.S.); xuyao0307@wust.edu.cn (Y.X.)

**Keywords:** chimeric antigen receptor T cells, CD99, dynamic cultivation, oxidative phosphorylation, TCA cycle, functional persistence

## Abstract

The manufacturing process contributes significantly to the proliferation, metabolic state, and functional persistence of chimeric antigen receptor (CAR)-T cells. However, how different culture systems regulate CAR-T cell metabolism and thereby influence their long-term antitumor activity remains poorly understood. In this study, we compared dynamic cultivation using a wave bioreactor with static expansion systems (gas-permeable and conventional T-flasks) for the production of CD99-specific CAR-T cells. CAR-T cells expanded by the wave bioreactor exhibited faster proliferation and stronger cytotoxicity during culture. Upon repeated antigen stimulation, they retained these enhanced functional properties and showed the reduced expression of immune checkpoint molecules, preferentially preserved memory-like subsets, and displayed transcriptional features consistent with memory maintenance and exhaustion resistance. Targeted metabolomic profiling revealed enhanced Tricarboxylic Acid (TCA) cycle activity and features consistent with sustained oxidative phosphorylation, supporting mitochondrial-centered metabolic reprogramming. In a Ewing sarcoma xenograft model, wave bioreactor-cultured CAR-T cells showed a greater percentage of memory-like tumor-infiltrating lymphocytes. Collectively, these results indicate that wave bioreactor-based dynamic cultivation promotes mitochondrial metabolic reprogramming, which is characterized by an enhanced TCA cycle and sustained oxidative phosphorylation, thereby sustaining CAR-T cell functionality and providing a robust platform for the manufacturing of potent and durable cellular therapeutics.

## 1. Introduction

Chimeric antigen receptor (CAR)-engineered T cells (CAR-T cells) eliminate tumor cells by directly recognizing extracellular tumor-associated antigens, independently of major histocompatibility complex (MHC)-restricted antigen presentation [1,2]. A typical CAR consists of an extracellular antigen recognition domain and intracellular signaling domains that activate T cells upon antigen binding, triggering cytotoxic responses against tumor cells [3]. CD99 is highly expressed in Ewing sarcoma [4], making it an attractive additional target for therapy. In our previous work, we developed CD99-specific CAR-T cells that exhibited potent antitumor activity against various tumor types [2], while showing a favorable safety profile with only minimal cytotoxicity toward activated T cells during in vitro culture [2]. In the present study, this CAR-T model was employed to investigate how biomanufacturing environments regulate CAR-T cell metabolism and shape their functional properties.

Although CAR-T cell therapy has shown substantial progress in cancer treatment [5], its long-term antitumor efficacy remains hindered by several obstacles [6,7,8], with T cell exhaustion being a key driver of therapeutic failure. This dysfunctional state results from both intrinsic tonic signaling within CAR-T cells [9,10] and extrinsic pressures from the tumor microenvironment [10,11,12] and is characterized by impaired cytotoxic function and reduced persistence. Chronic antigen exposure drives memory-like CAR-T cells toward exhaustion, whereas the enrichment of Stem Cell-Like Memory T Cell (TSCM) or naïve subsets is associated with enhanced persistence and durable clinical responses [13,14].

Recent work has demonstrated that the manufacturing process can substantially influence key quality attributes of CAR-T cells. Medium formulation is an important contributor to this variability, as serum-free or animal component-free preparations improve batch consistency [15,16,17], and cytokines such as IL-7 and IL-15 have been shown to better support stem-like and memory-associated properties compared with IL-2 alone [16]. In addition to the medium composition, the physical culture environment also plays a critical role in shaping CAR-T cells’ phenotypes and functionality. Current manufacturing systems are broadly categorized into static and dynamic culture platforms. Static systems rely primarily on passive diffusion for gas exchange [18,19], whereas dynamic systems employ active mixing to regulate dissolved oxygen, pH, and nutrient distribution with greater precision [20,21,22]. Studies using stirred-tank bioreactors have demonstrated that improved oxygen and nutrient availability can markedly enhance CAR-T cell expansion, support a favorable CD4/CD8 composition, enrich memory-associated subsets, and reduce exhaustion [16,23]. In recent years, several platforms providing automated and closed manufacturing environments have been introduced, including systems such as CliniMACS Prodigy, Cocoon, microfluidic bioreactors, and wave bioreactors. These automated approaches help to reduce operator-dependent variability and support the more consistent regulation of culture parameters throughout CAR-T cell production [24,25,26,27,28]. Among them, microfluidic bioreactors integrate dynamic culture with automated real-time regulation; they have been reported to improve metabolic stability during CAR-T cell expansion, while maintaining antitumor activity at high cell densities [27]. In addition, tumor-infiltrating lymphocytes (TILs) expanded in wave bioreactors displayed a greater percentage of CD62L^+^ cells compared to those cultured in static bags [28]. However, how dynamic versus static cultivation regulates CAR-T cell metabolic programming and thereby influences their long-term antitumor activity remains incompletely elucidated, underscoring the importance of mechanistic studies to optimize CAR-T manufacturing.

In this study, we systematically compared dynamic cultivation using a wave bioreactor with static systems (gas-permeable flasks and T-flasks) for the preparation of CD99-targeted CAR-T cells. We found that CAR-T cells undergoing expansion in the wave bioreactor exhibited markedly enhanced proliferation and cytotoxicity. Metabolomic profiling revealed that dynamic cultivation promoted a metabolic program centered on mitochondrial function, characterized by increased tricarboxylic acid (TCA) cycle activity and features consistent with sustained oxidative phosphorylation. This metabolic advantage translated into improved cytotoxicity, a memory-like phenotype, and resistance to exhaustion under repeated antigen stimulation, providing mechanistic insights to guide the development of efficient and durable CAR-T cell manufacturing strategies.

## 2. Results

### 2.1. Dynamic Cultivation Augments the Expansion and Cytotoxic Potency of CD99 CAR-T Cells

**Figure 1:**
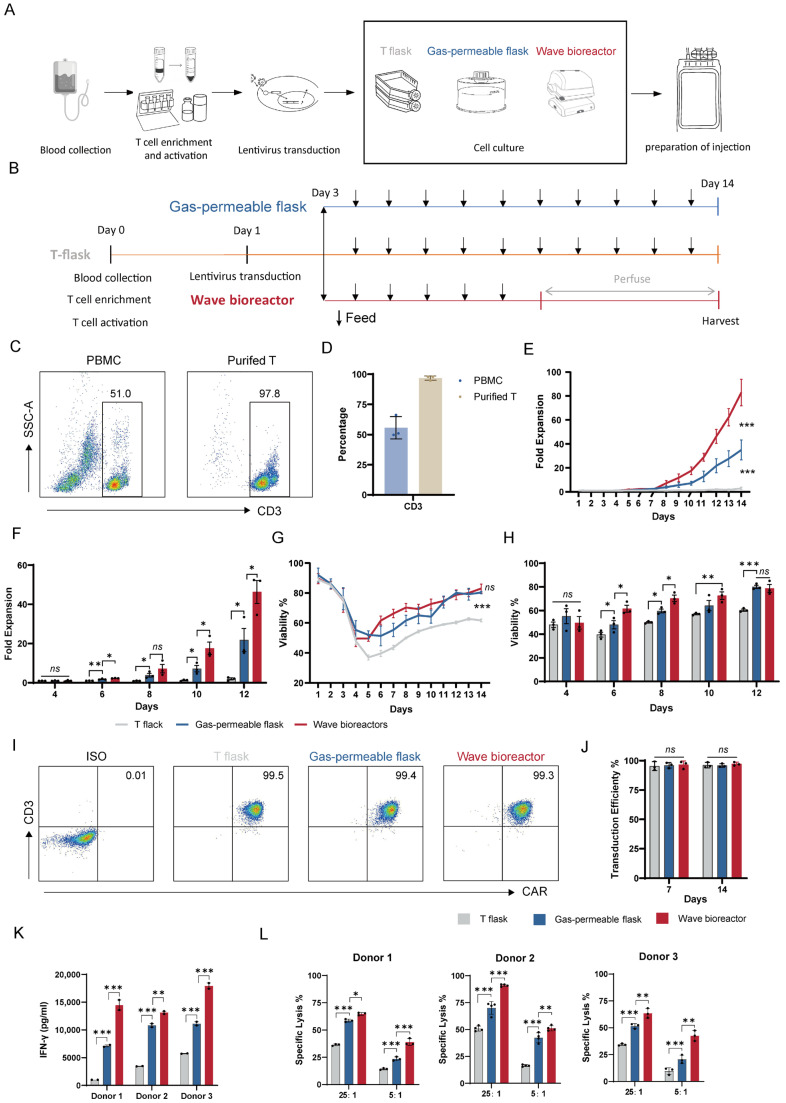
Effects of culture systems on the expansion and effector function of CD99 CAR-T cells. (**A**) Schematic illustration of a typical CAR-T cell therapy workflow. (**B**) Schematic overview of the 14-day CAR-T cell culture. (**C**) Representative flow plots showing frequencies of T cells before and after isolation. (**D**) Quantification of T cell percentages before and after isolation (n = 3 biologically independent replicates). (**E**) Expansion fold following transduction with CD99 CAR in different culture systems over 14 days (n = 3 biologically independent replicates). (**F**) T cell expansion fold following transduction with CD99 CAR in different culture systems on days 4, 6, 8, 10, and 12 (n = 3 biologically independent replicates). (**G**) Viability of CD99 CAR-T cells in different culture systems over 14 days (n = 3 biologically independent replicates). (**H**) Viability of total T cells transduced with CD99 CAR in different culture systems on days 4, 6, 8, 10, and 12 (n = 3 biologically independent replicates). (**I**) Representative flow plots showing frequencies of CD99 CAR-T cells on day 7 and day 14. (**J**) Fraction of CAR-positive cells assessed by flow cytometry on day 7 and day 14 (n = 3 biologically independent replicates). (**K**) Cytokine concentrations released by CD99 CAR-T cells following a 24 h co-culture with A-673 cells (from three independent donors) with an effector–to-target (E:T) ratio of 25:1 (n = 2 independent wells). (**L**) Tumor cell killing of A-673 cells after incubation with CD99 CAR-T cells for 3 h (from three independent donors) at E:T ratios of 25:1 and 5:1 (n = 3–4 independent wells). Data are presented as mean ± standard error of the mean (SEM). Statistical analysis was performed using two-way analysis of variance (ANOVA) followed by Šídák’s multiple comparisons test (**E**,**G**,**J**–**L**) or unpaired Student’s *t*-test (**F**,**H**). Statistical significance is indicated as follows: * *p* < 0.05, ** *p* < 0.01 and *** *p* < 0.001; ns, not significant.

To investigate how the cultivation mode influences CAR-T cell manufacturing, we expanded CD99-specific CAR-T cells under static conditions (using a T-flask and gas-permeable flask) and dynamic conditions (using a wave bioreactor) (Figure 1A). A standardized protocol for T cell isolation, activation, and lentiviral transduction was uniformly applied across all experimental groups (Figure 1B). All T cells used for lentiviral transduction exhibited purity greater than 95% (Figure 1C,D). Following the optimization of the bioreactor’s rocking parameters (Supplementary Figure S1A–C), we proceeded with comparative analyses.

CD99 CAR-T cells expanded by the wave bioreactor exhibited markedly superior proliferation compared to those cultured under static conditions (Figure 1E,F). During the expansion phase, the CAR-T cell population underwent a transient self-elimination process, characterized by an initial decrease in cell viability followed by recovery. Notably, the wave bioreactor significantly accelerated the restoration of cell viability (Figure 1G,H). The transduction efficiency was similar among all groups, with CAR expression approaching 100% at both day 7 and day 14, in agreement with our previous findings [2] (Figure 1I,J).

We next evaluated the functional impact of the cultivation mode on antitumor potency. Following antigen stimulation, IFN-γ secretion was highest in the wave bioreactor-cultivated group, moderate in the gas-permeable flask group, and lowest in the T-flask group (Figure 1K). Consistent with this observation, cytolytic assays demonstrated that wave bioreactor-expanded CAR-T cells achieved the most efficient target cell lysis (Figure 1L).

Collectively, these findings demonstrate that dynamic cultivation enhances both the expansion potential and cytotoxic functionality of CD99 CAR-T cells.

### 2.2. Dynamic Cultivation and Static Cultivation by Gas-Permeable Flask Similarly Reduce Exhaustion and Enrich Memory-like Subsets in CD99 CAR-T Cells

The antitumor efficacy of CAR-T cells is determined not only by their immediate cytolytic capacity but also by the preservation of favorable differentiation states that sustain long-term functionality. To investigate how dynamic and static culture systems influence the phenotypes of CD99 CAR-T cells, surface marker expression was assessed on day 14.

**Figure 2:**
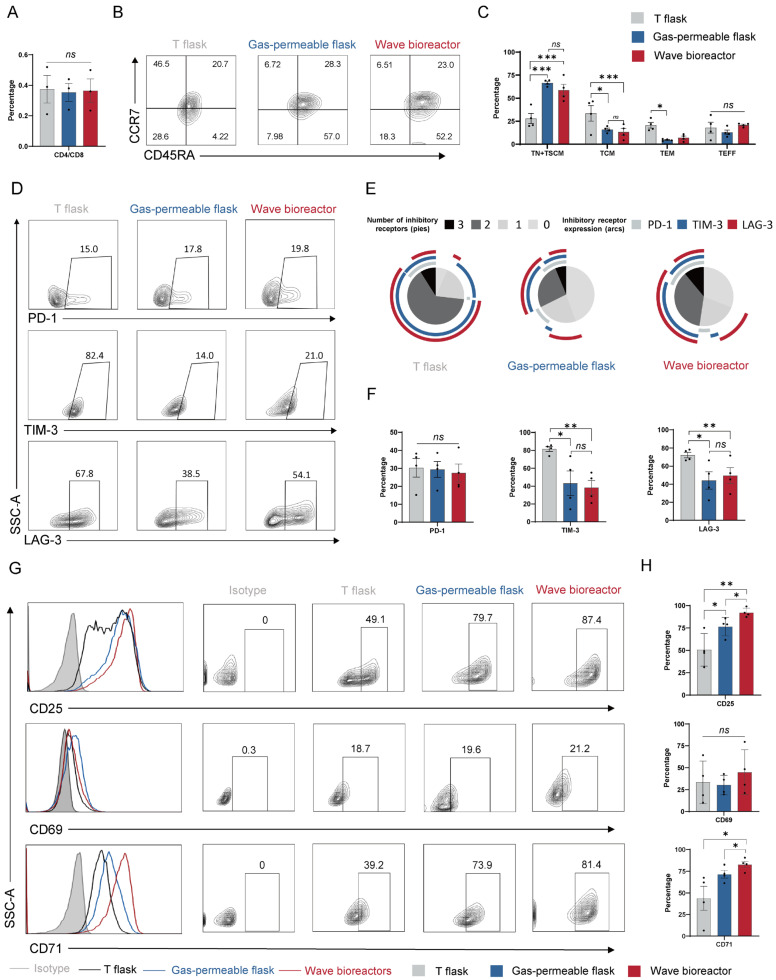
Effects of culture systems on the phenotypes of CD99 CAR-T cells. (**A**) Quantification of CD4^+^/CD8^+^ ratio of CAR-T cells (n = 3 biologically independent replicates). (**B**) Representative flow plots showing frequencies of CD99 CAR-T cells expressing CCR7 and CD45RA. (**C**) Quantification of CD99 CAR-T cells expressing memory markers (n = 4 biologically independent replicates). (**D**) Representative flow plots showing frequencies of CD99 CAR-T cells positive for exhaustion markers PD-1, TIM-3, and LAG-3. (**E**) Simplified Presentation of Incredibly Complex Evaluations (SPICE) analysis of inhibitory receptor expression in CD99 CAR-T cells. Representative CAR-T cells positive for exhaustion markers PD-1, TIM-3, and LAG-3 resulting from one of three independent donors. (**F**) Quantification of CD99 CAR-T cells expressing exhaustion markers (n = 4 biologically independent replicates). (**G**) Representative flow plots showing frequencies of CD99 CAR-T cells expressing activation markers CD25, CD69, and CD71. (**H**) Quantification of CD99 CAR-T cells displaying activation markers (n = 4 biologically independent replicates). Data are presented as mean ± SEM. Statistical analysis was performed using unpaired Student’s *t*-test (**F**,**H**), one way ANOVA followed by Šídák’s multiple comparisons test (**A**), or two-way ANOVA followed by Šídák’s multiple comparisons test (**C**). Statistical significance is indicated as follows: * *p* < 0.05, ** *p* < 0.01 and *** *p* < 0.001; ns, not significant.

From the perspective of the CD4/CD8 T cell phenotype, CD99-targeting CAR-T cells expanded under dynamic culture and in both static culture systems showed no significant differences (Figure 2A). However, for the memory and exhaustion phenotypes, both the wave bioreactor culture and static expansion in gas-permeable flasks led to a marked increase in naïve (TN) and stem cell memory (TSCM) subsets, along with a reduction in TIM-3^+^ and LAG-3^+^ cells, compared with the conventional T-flask culture, whereas PD-1 expression remained similar across all groups (Figure 2B–F). For T cell activation, wave bioreactor cultivation resulted in the highest expression levels of CD25 and CD71, while both the wave bioreactor and gas-permeable flasks yielded significantly higher expression than cultivation in T-flasks (Figure 2G,H). In contrast, no significant differences were detected in the expression of the early activation marker CD69 across all groups.

Although CD99 CAR-T cells expanded in the wave bioreactor and gas-permeable flasks exhibited largely similar CD4/CD8 ratios, memory profiles, and exhaustion states, the culture system may exert systematic effects on cellular transcriptional and metabolic programs. Therefore, we performed RNA-seq analysis on CAR-T cells expanded under both conditions to comprehensively evaluate the impacts of different biomanufacturing environments on cellular molecular features.

### 2.3. RNA-seq Analysis Reveals Dynamic Cultivation-Induced Reprogramming of Cytotoxic and Metabolic Programs

**Figure 3:**
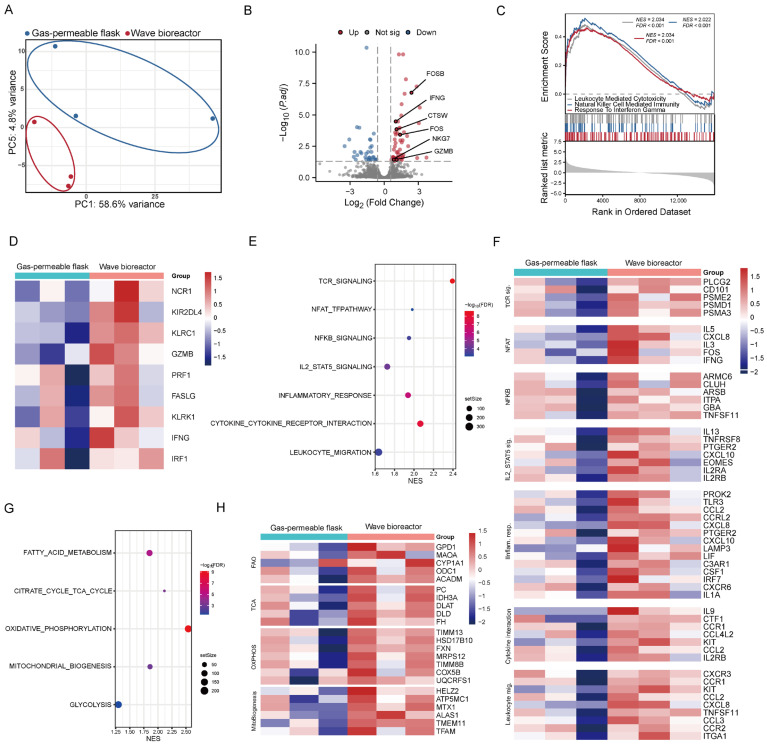
Effects of culture systems on the transcriptomic profile of CD99 CAR-T cells. (**A**) Gene expression data analyzed by principal component analysis (PCA). Points represent individual donors. (**B**) Volcano plot showing differentially expressed genes between CD99 CAR-T cells cultured in gas-permeable flasks and a wave bioreactor from three independent donors. Genes with an adjusted *p*-value < 0.05 and fold change ≥ 0.58 were considered significant. (**C**) Representative gene set enrichment analysis (GSEA) enrichment plot of effector function-related pathways. (**D**) Heat map showing expression profiles of selected representative genes from pathways identified in (**C**). (**E**) Bubble plot showing representative GSEA enrichment of immune activation and signaling-related pathways. (**F**) Heat map showing expression profiles of selected representative genes from pathways identified in (**E**). (**G**) Bubble plot showing representative GSEA enrichment of metabolic and mitochondrial function-related pathways. (**H**) Heat map showing expression profiles of selected representative genes from pathways identified in (**G**).

To characterize the molecular features associated with different cultivation systems, RNA sequencing was performed on CD99 CAR-T cells expanded in the wave bioreactor and in gas-permeable flasks. Principal component analysis (PCA) revealed clear segregation between CAR-T cells expanded in the two groups, underscoring the profound transcriptomic reprogramming induced by the distinct cultivation modes (Figure 3A). Differential expression analysis identified 58 genes that were significantly upregulated and 33 that were downregulated under wave bioreactor cultivation (Figure 3B). Strikingly, the upregulated gene set was highly enriched for markers of effector T cell function, including cytolytic molecules (*GZMB*, *NKG7*), a key effector cytokine (*IFNG*), and a granule-associated protease (*CTSW*). Notably, we also observed the concomitant induction of immediate early genes *FOS* and *FOSB*, which are rapidly transcribed upon T cell receptor stimulation and can form the AP-1 transcription complex, a pivotal regulator of T cell activation and differentiation.

To define the molecular basis of the enhanced cytotoxicity, we performed gene set enrichment analysis (GSEA). This revealed a coherent transcriptional signature in wave bioreactor-cultivated CAR-T cells, dominated by the significant enrichment of immune effector programs such as leukocyte-mediated cytotoxicity, natural killer cell-mediated immunity, and response to interferon-γ (Figure 3C). This program was operationalized by the coordinated upregulation of a suite of genes encoding the essential machinery for target cell recognition (*NCR1*, *KLRK1*), pore-forming execution (*PRF1*), and apoptotic signaling (*FASLG*), as visualized by heatmap analysis (Figure 3D). Thus, dynamic cultivation endows CAR-T cells with potent and multifaceted cytotoxic response capabilities at the transcriptional level.

Beyond the core cytolytic programs, wave bioreactor-cultivated CAR-T cells exhibited the coordinated upregulation of auxiliary pathways that functionally support effector responses. GSEA indicated that these cells are primed for sustained activation through enhanced TCR, NFAT, and NFKB signaling and promote continuous proliferation via the IL2/STAT5 signaling pathway. Furthermore, they displayed an enriched capacity for microenvironmental crosstalk via inflammatory response and cytokine–receptor interaction pathways, alongside increased potential for tissue infiltration through leukocyte migration programs (Figure 3E). This multifaceted supportive state was reflected at the molecular level by the concerted upregulation of key regulators: from early activation (*PLCG2*), sustained activation (*ARMC6*, *CLUH*, *TNFSF11*), and proliferation signals (*IL2RA/B*) to the expression of diverse cytokines and chemoattractants (*IL9*, *IL5*, *CCL2*, *CCL3*) that can shape the immune response and pattern recognition receptors (*TLR3*) that may bolster inflammatory tone (Figure 3F).

We next investigated whether the enhanced effector profile was supported by a favorable metabolic state. Notably, metabolic gene set analysis revealed the fundamental rewiring of energy metabolism in CAR-T cells expanded in the wave bioreactor, characterized by the coordinated upregulation of oxidative programs—including oxidative phosphorylation (OXPHOS), the tricarboxylic acid (TCA) cycle, fatty acid oxidation (FAO), and mitochondrial biogenesis—while glycolysis showed only modest upregulation (Figure 3G). Among these pathways, the TCA cycle and OXPHOS exhibited the highest normalized enrichment scores (NESs) and represent the core components of mitochondrial metabolism. This shift was further supported by the elevated expression of key genes involved in TCA cycle function (*IDH3A*, *FH*), mitochondrial assembly and function (*FXN*, *MTX1*, *TIMM13*), and metabolic adaptation (*GPD1*) (Figure 3H). Collectively, these data define a transition toward an oxidative metabolic phenotype, which is characteristic of long-lived, metabolically flexible memory T cells.

Collectively, these data indicate that dynamic cultivation is associated with broad metabolic reprogramming in CD99 CAR-T cells. The coordinated enrichment of oxidative phosphorylation and the TCA cycle suggests the establishment of a metabolically robust state that may support the energetic and biosynthetic demands required for sustained T cell activity. Within this metabolic context, CAR-T cells expanded in the wave bioreactor exhibited the enhanced transcriptional activation of cytotoxic and effector programs. To further validate whether the metabolic and functional features observed at the transcriptional level were reflected in the actual cellular state, we subsequently performed metabolic assessments on the CAR-T cells.

### 2.4. Functional Metabolic Validation Confirms Enhanced Mitochondrial Oxidative Metabolism in Dynamically Cultivated CD99 CAR-T Cells

**Figure 4:**
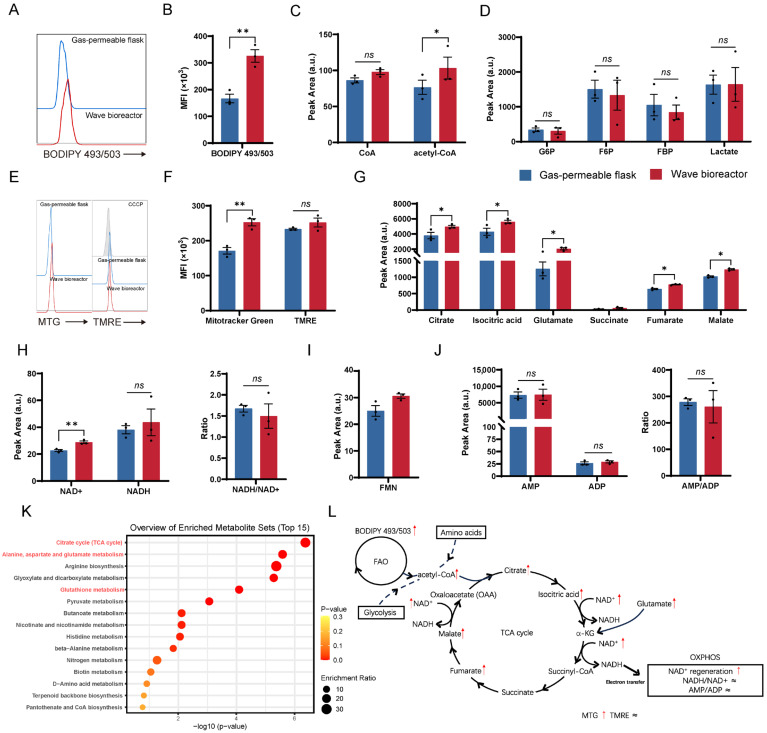
Metabolic characteristics of CAR-T cells under dynamic culture conditions. (**A**) Representative flow plots showing BODIPY 493/503 fluorescence intensity for CD99 CAR-T cells. (**B**) Quantification of CD99 CAR-T cell BODIPY 493/503 fluorescence intensity (n = 3 biologically independent replicates). (**C**) Quantification of CoA and acetyl-CoA peak areas in CD99 CAR-T cells (n = 3 biologically independent replicates). (**D**) Quantification of key glycolytic metabolites in CD99 CAR-T cells (n = 3 biologically independent replicates). (**E**) Representative flow plots showing MitoTracker Green (MTG) and Tetramethylrhodamine Ethyl Ester (TMRE) fluorescence intensity for tumor-infiltrating CAR-T cells. (**F**) Quantification of tumor-infiltrating CAR-T cells’ MTG and TMRE fluorescence intensity (n = 3 biologically independent replicates). (**G**) Quantification of key TCA cycle metabolites in CD99 CAR-T cells (n = 3 biologically independent replicates). (**H**–**J**) Quantification of key redox and energy metabolites in CD99 CAR-T cells (n = 3 biologically independent replicates). (**K**) KEGG (Kyoto Encyclopedia of Genes and Genomes) pathway enrichment analysis of differentially abundant metabolites in CD99 CAR-T cells. (**L**) Overview of key metabolic pathways in CD99 CAR-T cells. Upward arrows (↑) indicate upregulation, whereas “≈” indicates no significant difference compared with the control. Data are presented as mean ± SEM. Statistical analysis was performed using unpaired Student’s *t*-test (**B**,**F**) or paired Student’s *t*-test (**C**,**D**,**G**–**J**). Statistical significance is indicated as follows: * *p* < 0.05 and ** *p* < 0.01; ns, not significant.

To experimentally validate the metabolic programs inferred from transcriptomic analysis, we performed a series of functional and targeted metabolic assays in CAR-T cells expanded under wave bioreactor or gas-permeable flask conditions. Based on the enrichment of fatty acid oxidation (FAO)-related gene sets in the RNA-seq analysis, we first assessed intracellular neutral lipid content using BODIPY 493/503 staining. CAR-T cells expanded in the wave bioreactor exhibited a significantly increased BODIPY 493/503 signal compared with those expanded in gas-permeable flasks (Figure 4A,B), indicating enhanced lipid accumulation. Consistently, targeted metabolomic profiling revealed a concomitant increase in acetyl-CoA levels in the wave bioreactor group (Figure 4C), supporting elevated lipid-derived carbon entry into mitochondrial metabolism. Meanwhile, the targeted analysis of key glycolytic intermediates showed no significant differences between the two groups (Figure 4D), suggesting that glycolytic flux is comparable under both culture conditions.

Given the prominent enrichment of mitochondrial metabolic pathways, we further evaluated the mitochondrial mass and membrane potential. Flow-cytometric analysis using MitoTracker Green (MTG) revealed a marked increase in mitochondrial content in wave bioreactor-expanded CAR-T cells, whereas the mitochondrial membrane potential, assessed by tetramethylrhodamine ethyl ester (TMRE) staining with carbonyl cyanide m-chlorophenyl hydrazone (CCCP) as a negative control, remained comparable between the two groups (Figure 4E,F). These findings indicate the expansion of the mitochondrial mass without evidence of mitochondrial hyperpolarization or dysfunction.

Targeted metabolomic profiling of the tricarboxylic acid (TCA) cycle demonstrated the coordinated elevation of multiple intermediates, including citrate, isocitric acid, fumarate, and malate, in CAR-T cells expanded in the wave bioreactor (Figure 4G). In parallel, glutamate, as a key anaplerotic substrate contributing to α-ketoglutarate production, was also significantly increased, further supporting enhanced TCA cycle activity.

The assessment of redox and energetic cofactors revealed a significant increase in nicotinamide adenine dinucleotide (oxidized form) (NAD^+^) levels in the wave bioreactor group, while the nicotinamide adenine dinucleotide (reduced form) (NADH) abundance and the NADH/NAD^+^ ratio remained unchanged (Figure 4H). This pattern is consistent with efficient NAD^+^ regeneration and sustained electron flow through the oxidative phosphorylation machinery. In line with this interpretation, flavin mononucleotide (FMN), a cofactor associated with the electron transport chain, exhibited an upward trend, although it did not reach statistical significance (Figure 4I). In contrast, adenosine monophosphate (AMP), adenosine diphosphate (ADP), and the AMP/ADP ratio showed no significant differences between groups (Figure 4J), indicating that cellular energy homeostasis was maintained.

We further performed pathway enrichment analysis based on differentially abundant metabolites. The tricarboxylic acid (TCA) cycle emerged as a significantly enriched pathway, accompanied by the enrichment of alanine, aspartate, and glutamate metabolism, as well as pyruvate metabolism (Figure 4K). These findings underscore the coordinated remodeling of mitochondrial-centered metabolic networks.

Collectively, these data provide functional and metabolic evidence that wave bioreactor cultivation promotes enhanced mitochondrial metabolic activity in CD99 CAR-T cells, characterized by an increased mitochondrial mass, elevated TCA cycle flux, and sustained oxidative phosphorylation capacity, while maintaining energetic stability (Figure 4L).

### 2.5. Metabolomic Reprogramming of the TCA Cycle and OXPHOS Confers Functional Persistence and Resistance to Exhaustion in CD99 CAR-T Cells upon Repeated Antigen Challenge

**Figure 5:**
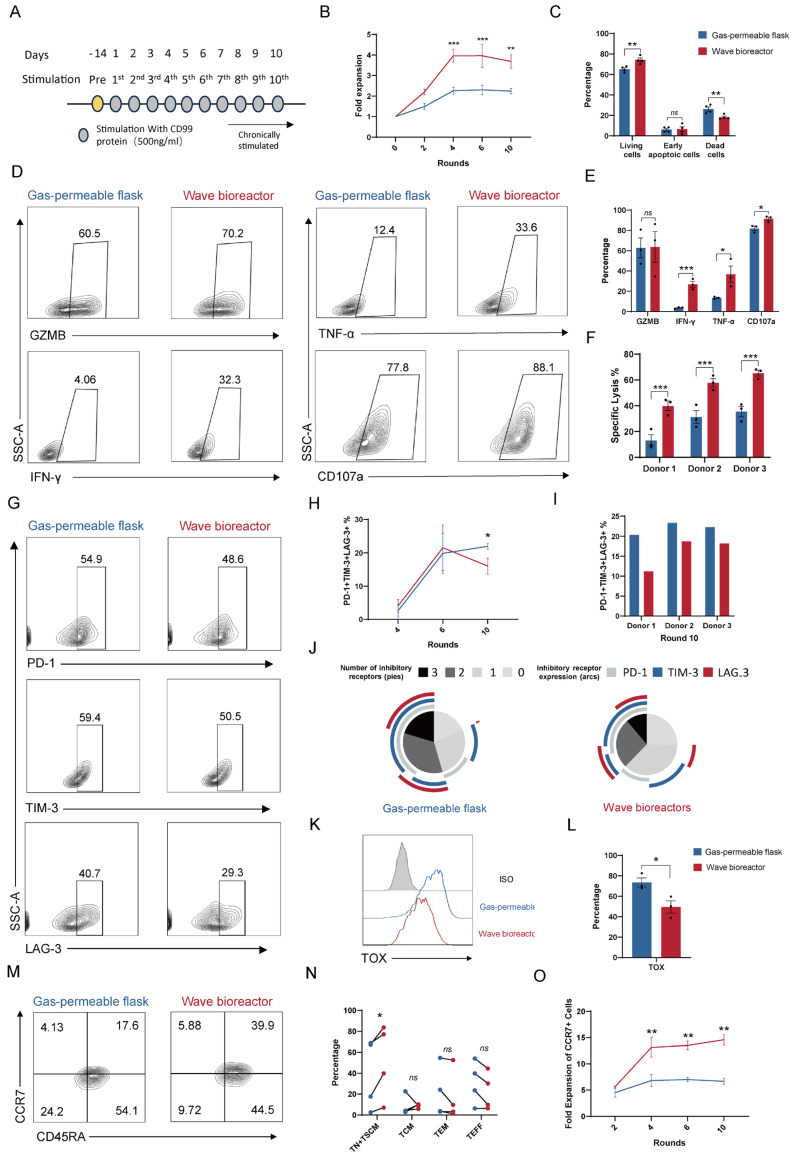
Effects of culture systems on the long-term cytotoxic activity of CD99 CAR-T cells. (**A**) Schematic illustration of the in vitro multi-round antigen stimulation protocol. (**B**) CD99 CAR-T cell expansion fold after multiple rounds of CD99 protein stimulation (n = 3 biologically independent replicates). (**C**) Proportions of living, early apoptotic, and dead CD99 CAR-T cells after repeated stimulation with CD99 protein for ten rounds (n = 3 biologically independent replicates). (**D**) Representative flow plots showing frequencies of CD99 CAR-T cells positive for GZMB, TNF-α, IFN-γ, and CD107a after ten rounds of stimulation. (**E**) Quantification of CD99 CAR-T cells positive for GZMB, TNF-α, IFN-γ, and CD107a after repeated stimulation with CD99 protein for ten rounds (n = 3 biologically independent replicates). (**F**) Tumor cell killing of A-673 cells after incubation with CD99 CAR-T cells for 3 h (from three independent donors) after ten rounds of stimulation at E:T ratios of 0.5:1 (n = 3 independent wells). (**G**) Representative flow plots showing CD99 CAR-T cells positive for exhaustion markers PD-1, TIM-3, and LAG-3 after ten rounds of stimulation. (**H**) SPICE analysis of inhibitory receptor expression in CD99 CAR-T cells after ten rounds of stimulation. Representative result from one of three independent donors. (**I**,**J**) Quantification of CD99 CAR-T cells co-positive for PD-1, TIM-3, and LAG-3 after ten rounds of stimulation (n = 3 biologically independent replicates). (**K**) Representative flow plots showing CD99 CAR-T cells positive for TOX after ten rounds of stimulation. (**L**) Quantification of CD99 CAR-T cells positive for TOX after ten rounds of stimulation (n = 3 biologically independent replicates). (**M**) Representative flow plots showing CD99 CAR-T cells positive for CCR7 and CD45RA after ten rounds of stimulation. (**N**) Quantification of CD99 CAR-T cells positive for memory markers after ten rounds of stimulation (n = 3 biologically independent replicates). (**O**) Fold expansion of CCR7^+^ memory CD99 CAR-T cells after multiple rounds of stimulation (n = 3 biologically independent replicates). Data are presented as mean ± SEM. Statistical analysis was performed using two-way ANOVA followed by Šídák’s multiple comparisons test (**B**,**C**,**F**,**I**,**N**,**O**) or unpaired Student’s *t*-test (**E**,**L**). Statistical significance is indicated as follows: * *p* < 0.05, ** *p* < 0.01 and *** *p* < 0.001; ns, not significant.

To functionally validate the impact of the metabolic programs revealed by metabolomics, we challenged CD99 CAR-T cells from dynamic cultivation (using a wave bioreactor) and static cultivation (using gas-permeable flasks) with 10 rounds of antigen stimulation by plate-bound recombinant CD99 protein (Figure 5A). Plate-bound protein provides standardized and reproducible stimulation, minimizing variability in antigen density, target cell viability, and effector-to-target ratios during multiple rounds of antigen stimulation, although this approach has certain limitations in fully recapitulating the tumor microenvironment.

Wave bioreactor-derived CAR-T cells exhibited a faster expansion rate during the first four stimulation cycles (Figure 4B). Although both groups ceased expansion beyond the fourth round, the cumulative fold expansion of wave bioreactor-expanded CAR-T cells remained significantly higher throughout the entire stimulation period (Figure 5B). This enhanced proliferative capacity culminated in a larger fraction of viable cells after the final round, confirming the functional benefit of dynamic culturing-driven expansion on T cell longevity (Figure 5C).

Following multiple rounds of antigen challenge, wave bioreactor-expanded CAR-T cells exhibited functionally superior effector responses. Compared to gas-permeable flask-cultured cells, they produced significantly more IFN-γ and TNF-α and displayed enhanced CD107a surface expression, indicative of robust cytokine secretion and degranulation (Figure 5D,E). This augmented cytokine production translated into consistently stronger target cell killing at low effector-to-target ratios across multiple donors, despite comparable levels of GZMB (Figure 5D–F). Thus, the dynamic culture system endows CAR-T cells with a sustained antitumor profile.

Prolonged antigen exposure revealed a critical divergence in exhaustion states. While the frequency of PD-1^+^TIM-3^+^LAG-3^+^ cells was comparable at earlier time points, wave bioreactor-expanded CAR-T cells exhibited a significantly lower burden of this triple-positive exhausted population after 10 stimulation rounds (Figure 5G–I). Simplified Presentation of Incredibly Complex Evaluations (SPICE) analysis confirmed a decrease in the expression of inhibitory checkpoints in the wave bioreactor group (Figure 5J). At the molecular level, this resistant phenotype was characterized by significantly lower expression of the exhaustion-associated transcription factor TOX (Figure 5K,L). Thus, the dynamic culture system mitigates the development of terminal exhaustion under multiple rounds stimulation.

In terms of the memory phenotype, after 10 rounds of stimulation, wave bioreactor-expanded CAR-T cells exhibited a greater percentage of TN + TSCM cells (Figure 5M,N), and their CCR7^+^ memory-like cells showed improved expansion compared to those in gas-permeable flask cultures (Figure 5O).

Collectively, these functional assays indicate that the enhanced TCA cycle activity and sustained oxidative phosphorylation conferred by dynamic cultivation are associated with improved functional persistence, resistance to exhaustion, and maintenance of memory potential in CD99 CAR-T cells under chronic antigen challenge.

### 2.6. Dynamic Cultivation Confers a Transcriptionally Resilient State That Sustains Effector Function Under Chronic Antigen Challenge

**Figure 6:**
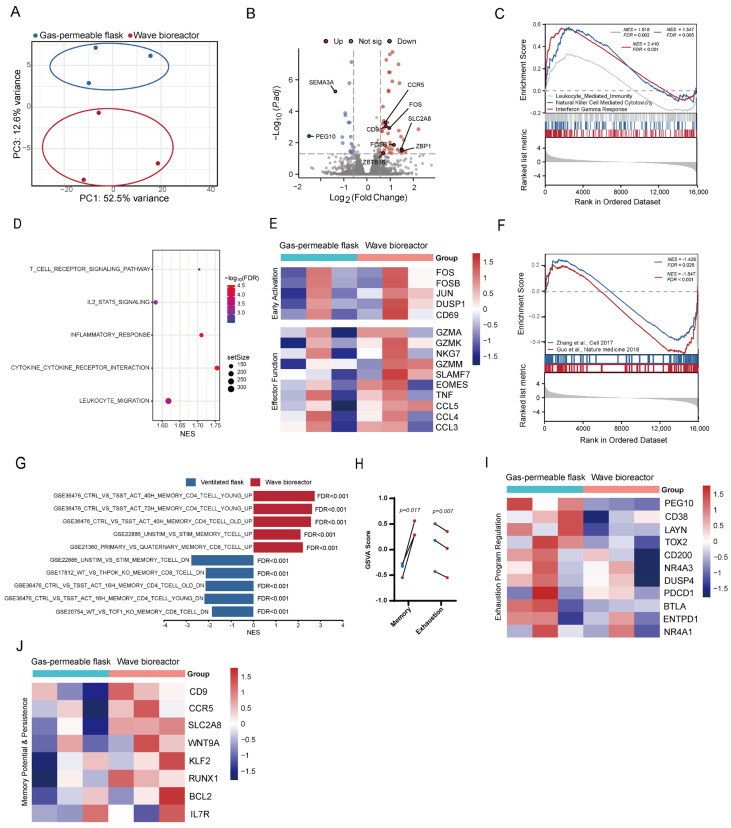
Effects of culture systems on the transcriptome profile of CD99 CAR-T cells after repeated stimulation. (**A**) Gene expression data analyzed by principal component analysis (PCA). Points represent individual donors. (**B**) Volcano plot showing variably expressed genes between CD99 CAR-T cells cultured in gas-permeable flasks and wave bioreactors from three independent donors. Genes with an adjusted *p*-value < 0.05 and fold change ≥ 0.58 were considered significant. (**C**) Representative GSEA enrichment plot of effector function-related pathways. (**D**) Bubble plot showing representative GSEA enrichment of immune activation and signaling-related pathways. (**E**) Heat map showing expression profiles of selected representative genes from pathways identified in (**C**,**D**). (**F**) GSEA analysis of exhaustion-related gene sets. (**G**) Normalized enrichment scores (NESs) of significantly up- or downregulated gene sets in CD99 CAR-T cells cultured in gas-permeable flasks versus wave bioreactors, as determined by GSEA using MSigDB C7 gene sets. (**H**) Gene set variation analysis (GSVA) scores of memory- and exhaustion-associated gene signatures. (**I**) Heat map showing expression profiles of selected representative genes from pathways identified in (**F**). (**J**) Heat map showing expression profiles of selected representative genes from pathways identified in (**G**) [29,30].

After observing that CD99 CAR-T cells expanded via dynamic cultivation exhibited long-term functional persistence, we performed an RNA-seq analysis of the cells following 10 rounds of antigen stimulation to assess their transcriptional features and functional states under sustained stimulation. Principal component analysis revealed clear segregation between cells initially expanded under the wave bioreactor and those cultured in gas-permeable flasks, even after multiple rounds of antigen challenge (Figure 6A), indicating a durable molecular imprint induced by the cultivation mode. Differential expression analysis identified a distinct transcriptional signature in the wave bioreactor cultivation group, featuring 48 upregulated and 17 downregulated genes (Figure 6B).

Notably, this transcriptional state was characterized by the sustained enhancement of effector cell programs. Gene set enrichment analysis (GSEA) uncovered the significant enrichment of cytotoxicity-related pathways, including leukocyte-mediated immunity, natural killer cell-mediated cytotoxicity, and the interferon-gamma response (Figure 6C). Additionally, pathways supporting T cell effector activity—including T cell receptor signaling, IL-2/STAT5 signaling, and cytokine–cytokine receptor interaction—remained upregulated (Figure 6D). Consistent with these pathway-level findings, core genes linked to T cell activation (*FOS*, *JUN*, *CD69*) and effector function (*GZMA*, *NKG7*, *CCL5*) maintained elevated expression in dynamically cultivated cells following chronic stimulation (Figure 6E).

The ability to retain this functional capacity was supported by transcriptional programs that countered exhaustion and promoted cellular longevity. GSEA using well-validated exhaustion gene sets (Zhang et al., 2017 [29]; Guo et al., 2018 [30]) demonstrated the significant downregulation of exhaustion signatures in dynamically cultivated cells (Figure 6F). Furthermore, these cells exhibited a coordinated transcriptional shift favoring memory formation, marked by the upregulation of pathways positively regulating memory development and the downregulation of pathways that impede memory cell formation (Figure 6G). Quantitative gene set variation analysis (GSVA) scoring confirmed that dynamically cultivated cells achieved a state of significantly enhanced memory potential paired with reduced exhaustion following prolonged stimulation (Figure 6H). The gene sets used for GSVA scoring are listed in Supplementary Table S2. This phenotype was reflected in the coordinated upregulation of memory-promoting genes (*CD9*, *CCR5*) and the suppression of exhaustion markers (*SEMA3A*, *PEG10*) (Figure 6I,J).

In summary, dynamic cultivation via a wave bioreactor induces a transcriptionally resilient state in CD99 CAR-T cells, sustaining effector function, limiting exhaustion, and promoting memory formation even after repeated antigen stimulation.

### 2.7. Dynamic Cultivation During Expansion Enhances the Stem-like Memory Phenotype in Tumor-Infiltrating CD99 CAR-T Cells

**Figure 7:**
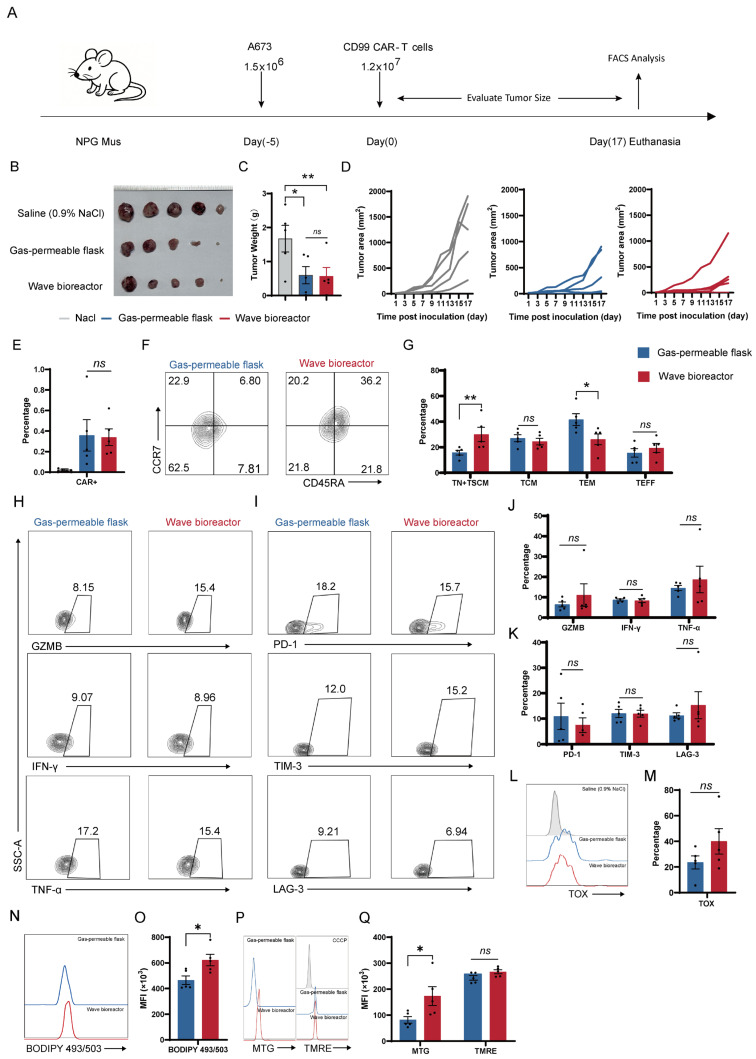
Effects of culture systems on the in vivo antitumor function of CD99 CAR-T cells. (**A**) Experimental timeline of the CDX (Cell line–derived xenograft) model. (**B**) Representative tumor images from each treatment group at the experimental endpoint. (**C**) Tumor weights measured on day 17 after i.v. administration of saline (0.9% NaCl) or CAR-T cells (n = 5 mice). (**D**) Tumor volume changes over time following i.v. administration of saline (0.9% NaCl), gas-permeable flask, and wave bioreactor treatments (n = 5 mice). (**E**) Quantification of tumor-infiltrating CAR-T cell percentage on day 17 after CAR-T cell infusion (n = 5 mice). (**F**) Representative flow plots showing CCR7 and CD45RA expression on CAR-T cells isolated from tumor tissue on day 17 after infusion. (**G**) Quantification of tumor-infiltrating CAR-T cells positive for memory-associated markers (n = 5 mice). (**H**) Representative flow plots showing GZMB, IFN-γ, and TNF-α expression for CAR-T cells isolated from tumor tissue on day 17 after infusion. (**I**) Representative flow plots showing PD-1, TIM-3, and LAG-3 positivity for CAR-T cells isolated from tumor tissue on day 17 after infusion. (**J**) Quantification of tumor-infiltrating CAR-T cells positive for GZMB, IFN-γ, and TNF-α (n = 5 mice). (**K**) Quantification of tumor-infiltrating CAR-T cells positive for exhaustion-associated markers (n = 5 mice). (**L**) Representative flow plots showing TOX positivity for tumor-infiltrating CAR-T cells on day 17 after infusion. (**M**) Quantification of tumor-infiltrating CAR-T cells positive for TOX (n = 5 mice). (**N**) Representative flow plots showing BODIPY 493/503 fluorescence intensity for tumor-infiltrating CAR-T cells on day 17 after infusion. (**O**) Quantification of tumor-infiltrating CAR-T cell BODIPY 493/503 fluorescence intensity. (**P**) Representative flow plots showing MTG andTMREfluorescence intensity for tumor-infiltrating CAR-T cells on day 17 after infusion. (**Q**) Quantification of tumor-infiltrating CAR-T cells’ MTG and TMRE fluorescence intensity. Data are presented as mean ± SEM. Statistical analysis was performed using unpaired Student’s *t*-test (**C**,**E**,**J**,**K**,**M**,**O**,**Q**) or two-way ANOVA followed by Šídák’s multiple comparisons test (**G**). Statistical significance is indicated as follows: * *p* < 0.05 and ** *p* < 0.01; ns, not significant.

To assess whether the superior memory phenotype and functional persistence observed in vitro could be translated to an in vivo setting, we evaluated the tumor-suppressive function and differentiation states of CD99 CAR-T cells in a subcutaneous A673 xenograft model. NPG (NOD.Cg-Prkdc^scid^ Il2rg^tm1^/Vst) mice bearing established tumors were treated with a single dose of CAR-T cells expanded under dynamic cultivation (wave bioreactor) or static cultivation (gas-permeable flask) or a saline control (Figure 7A).

Compared with the saline control group, both the wave bioreactor and gas-permeable flask cultivation groups exhibited significantly inhibited tumor growth (Figure 7B). Notably, no statistically significant difference in tumor volume or weight was observed between the two CAR-T treatment groups at this endpoint (Figure 7B–D). Consistently, comparable proportions of tumor-infiltrating CAR-T cells were detected in both groups (Figure 7E).

Despite comparable effector function, wave bioreactor-cultivated CAR-T cells exhibited a profoundly distinct composition within the tumor microenvironment. They displayed a significantly enriched population of naïve (TN) and stem cell memory (TSCM) subsets, coupled with a reduction in terminal effector memory (TEM) cells (Figure 7F,G).

An analysis of tumor-infiltrating lymphocytes (TILs) revealed that the effector function and exhaustion states were comparable between the two groups, as indicated by the similar levels of cytokine production (IFN-γ, TNF-α), cytolytic molecule expression (GZMB), and exhaustion marker expression (PD-1, TIM-3, LAG-3, TOX) (Figure 7H–M).

To further evaluate the impacts of different culture systems on the metabolic status of tumor-infiltrating CAR-T cells, lipid metabolism and mitochondrial features were analyzed in CAR-T cells isolated from tumor tissue. Compared with cells expanded in gas-permeable flasks, CAR-T cells generated in the wave bioreactor exhibited significantly higher neutral lipid content, as indicated by the increased BODIPY 493/503 fluorescence intensity (Figure 7N,O), reflecting an enhanced lipid metabolism capacity. With respect to mitochondrial parameters, CAR-T cells from the wave bioreactor group displayed a significantly increased mitochondrial mass, as assessed by MTG, whereas the mitochondrial membrane potential, measured by TMRE, did not differ significantly between the two groups (Figure 7P,Q). These findings indicate that dynamic cultivation increases the mitochondrial abundance without inducing excessive mitochondrial polarization. Overall, dynamic expansion supports the maintenance of metabolic fitness in tumor-infiltrating CAR-T cells.

This indicates that dynamic cultivation during ex vivo expansion promotes the preservation of a less differentiated, stem-like memory phenotype in vivo, accompanied by distinct metabolic features. Although this did not enhance short-term tumor control, it may confer superior long-term potential.

## 3. Discussion

In this study, we systematically explored CD99 CAR-T cells by dynamic versus static cultivation. Our results show that dynamic cultivation is associated with metabolic reprogramming toward mitochondrial-centered oxidative metabolism, characterized by enhanced tricarboxylic acid (TCA) cycle activity and sustained oxidative phosphorylation capacity. This reprogramming not only enhances proliferation and cytotoxic activity, but also confers durable antitumor functionalities to CAR-T cells, as reflected by superior expansion, a larger proportion of memory-like cells, reduced exhaustion, and sustained cytotoxic capacity when subjected to repeated antigen stimulation. Importantly, a large proportion of memory-like cells was also preserved within tumors in the NPG xenograft model.

Under dynamic culture conditions, CD99 CAR-T cells exhibited the pronounced accumulation of neutral lipids, suggesting that fatty acid-related metabolic pathways may be preferentially engaged. The end product of FAO, acetyl-CoA, directly fuels the tricarboxylic acid (TCA) cycle, thereby continuously supplying substrates for oxidative phosphorylation. Consistent with this metabolic framework, enhanced FAO is commonly associated with improved mitochondrial oxidative capacity and long-term T cell functionality [31]. In contrast, glycolytic metabolism is primarily linked to rapid T cell activation and short-term effector responses but is generally insufficient to support durable antitumor activity [32]. Accumulating evidence indicates that T cells with a strong reliance on glycolysis are more susceptible to functional exhaustion under conditions of chronic antigen stimulation and display limited persistence [33]. Although glycolysis can also contribute acetyl-CoA to the TCA cycle through the pyruvate dehydrogenase complex, our metabolomic analysis revealed no significant differences in glycolysis-associated metabolites, including lactate, between dynamically and statically cultured CAR-T cells. These findings suggest that, under dynamic culture conditions, acetyl-CoA availability is unlikely to be driven by enhanced glycolytic flux, but instead predominantly relies on FAO-mediated metabolic input. Together, these findings support a metabolic configuration in which FAO-driven mitochondrial respiration provides sustained bioenergetic support, enabling effective cytotoxic function while avoiding an excessive reliance on glycolysis and thereby favoring long-term persistence.

At the metabolic level, CAR-T cells expanded under dynamic culture conditions exhibited a globally enhanced tricarboxylic acid (TCA) cycle profile. Targeted metabolomics revealed the coordinated upregulation of multiple TCA intermediates, including citrate, fumarate, and malate, indicating an efficiently operating, high-flux TCA cycle rather than metabolic stalling at a specific node. This pattern contrasts the previously reported pathological accumulation of certain intermediates, such as fumarate, which has been linked to T cell exhaustion [34]. In parallel, NAD^+^ levels were significantly elevated without a corresponding increase in NADH, and no differences were observed in AMP or ADP levels between groups, indicating efficient electron transport activity and preserved cellular energy homeostasis. Consistent with these findings, dynamically cultured CAR-T cells displayed a significant increase in mitochondrial mass while maintaining a stable mitochondrial membrane potential. These results suggest that the greater mitochondrial abundance provides additional spatial capacity to accommodate the enhanced TCA cycle activity and sustained oxidative phosphorylation, thereby supporting a higher overall metabolic capacity without imposing excessive functional stress on individual mitochondria. The enhanced TCA-OXPHOS program induced by dynamic culture may be driven by improved oxygen availability [35,36]. Dynamic rocking has been shown to increase dissolved oxygen transfer and reduce localized hypoxia, thereby supporting mitochondrial oxidative phosphorylation and TCA cycle activity while limiting HIF-1α-driven glycolytic reprogramming [37,38].

Because activated T cells generally express low levels of CD99, transient fratricide may occur during the early expansion of CD99 CAR-T cells, resulting in an initial decrease in viability. As CD99-low T cells are eliminated, viability subsequently recovers and stabilizes at approximately 80%. Preclinical studies and a clinical case of Ewing sarcoma indicate that this early fratricide does not impair antitumor activity [2,39]. In this context, the faster viability recovery observed under dynamic culture conditions may be supported by an efficient TCA-OXPHOS-based metabolic program, suggesting a potential strategy to optimize the manufacturing of fratricide-prone CAR-T cells.

The long-term therapeutic efficacy of CAR-T cells hinges on their ability to resist exhaustion and maintain a memory-functional population [40,41]. Our results confirm that dynamic cultivation grants a distinct advantage when cells face chronic antigen exposure. After multiple rounds of stimulation, dynamically cultivated cells retained potent cytotoxicity, showed reduced expression of exhaustion markers, and had a higher frequency of memory-associated populations. This functional robustness is further supported by a pro-memory and anti-exhaustion transcriptional signature. Importantly, the sustained OXPHOS state driven by dynamic cultivation has been shown to be inherently tied to the development and maintenance of memory T cells [42,43]. Previous studies have demonstrated that CAR-T cells incorporating a 4-1BB costimulatory domain, compared with those containing a CD28 domain, activate the AMPK-PGC-1α axis, thereby promoting mitochondrial biogenesis and FAO, enhancing memory-like features, and reducing exhaustion marker expression [10]. Similarly, metabolic interventions that promote OXPHOS—such as IL-15 supplementation [44], AMPK activators [45], or PGC-1α overexpression [46]—have been shown to extend CAR-T cell persistence, maintain effector function, and delay exhaustion under chronic stimulation. Thus, the metabolic programming induced by dynamic cultivation offers a plausible mechanistic explanation for the preserved memory-like phenotype and enhanced durability of these cells.

Our in vivo experiments further underscored the translational relevance of these observations, even though dynamic and static cultivation using gas-permeable flasks yielded comparable tumor control. This result was likely influenced by the characteristics of the NPG mouse system, which lacks host immune support, as well as by the aggressive nature of the xenografted tumors. Together, these factors may restrict the time window during which advantages related to memory differentiation can be fully manifested. Despite similar antitumor efficacy, dynamically cultivated cells showed a significantly enriched TN/TSCM subset and a reduced TEM population within the tumor microenvironment. These findings highlight that the metabolic programming established during ex vivo expansion may persist in vivo, favoring the preservation of a less differentiated, stem-like state—one associated with long-term persistence and a self-renewal capacity. Future clinical studies are warranted to validate the persistence and therapeutic relevance of these metabolic adaptations in patients.

Enhanced gas exchange in dynamic cultivation systems is frequently noted as a major advantage [22,35,47]. However, beyond oxygen availability, the mechanical forces generated by rocking motion may also influence CAR-T cell biology, and we also detected the concurrent upregulation of mechanosensitive signaling molecules YAP1, LATS1, and RAC1, suggesting that rocking-induced fluid shear stress and mechanical forces may add an extra layer of regulation to promote a favorable cell state. The loss of mechanosensitive transcription factors like YAP1 has been reported to impair cell migration and proliferation on stiff substrates [48]. Moreover, CAR-T cells exposed to mechanical stimulation exhibit enhanced responsiveness upon subsequent antigen challenges [49]. These observations suggest that mechanical cues may represent an additional regulatory mechanism underlying the more favorable functionality of dynamic cultivated CAR-T cells, a hypothesis that merits further investigation.

## 4. Materials and Methods

### 4.1. Cell Lines

The A673 Ewing’s sarcoma cell line was purchased from the Cell Bank of the Chinese Academy of Sciences (Shanghai, China). Cells were cultured in Dulbecco’s Modified Eagle Medium (DMEM) supplemented with 10% fetal bovine serum (FBS) and maintained in a humidified incubator set at 37 °C with a 5% CO_2_ atmosphere. For the generation of A673 cell line derivatives, a lentiviral expression vector was constructed to drive Luciferase expression under the transcriptional control of the eukaryotic translation elongation factor 1 alpha (EF-1α) promoter, which also contained a puromycin resistance gene as the selection marker. A673 cells were transduced with this recombinant lentivirus to establish the A673-LUCI cell line. Subsequent maintenance of A673-LUCI cells was performed in DMEM containing 10% FBS and puromycin.

### 4.2. CD99 CAR Lentiviral Vector Construction

The CD99-specific CAR lentiviral vector was kindly provided by Wuhan Bio-Raid Biotechnology Co., Ltd. (Wuhan, China). The CAR construct comprises a single-chain variable fragment (scFv) targeting CD99 (clone 12E7), followed by a CD8α hinge region, a CD28 transmembrane domain, and intracellular signaling domains from CD28 and 4-1BB, as well as the CD3ζ activation domain [2].

### 4.3. CAR-T Cell Culture

Peripheral blood mononuclear cells (PBMCs) were obtained from healthy donors through Ficoll density gradient centrifugation (GE Healthcare, Uppsala, Sweden). CD3^+^ T cells were subsequently purified via MACS^®^ human CD3 microbeads (Miltenyi Biotec, Bergisch Gladbach, Germany), following the standard protocol provided by the manufacturer. For activation, T cells were treated with TransAct™ reagent (Miltenyi Biotec, Gaithersburg, MD, USA) for 24 h; thereafter, they were transduced with CD99 chimeric antigen receptor (CAR) lentivirus at a multiplicity of infection (MOI) of 5, in the presence of 5 µg/mL polybrene (Yeasen, Shanghai, China). To avoid potential variability caused by the lentivirus, all batches of CD99 CAR-T cells in this study were generated using the same lot of lentiviral vectors with identical titers. On the next day (defined as day 2), the cells were divided and transferred into one of three distinct culture systems: a T-flask (original, details provided below), a gas-permeable flask (details provided below), or a wave bioreactor (details provided below). All CAR-T cells were cultured in TexMACS medium, which was supplemented with HEPES (InvivoGen, San Diego, CA, USA), 1000 IU/mL recombinant human interleukin-2 (IL-2; Novoprotein, Suzhou, China), and L-glutamine (Gibco, Grand Island, NY, USA).

### 4.4. T-Flask Culture

Cells were cultured at a density of 1.2–2 × 10^6^ cells/mL into T175 flasks (Corning, Corning, NY, USA). Fresh medium was supplemented on a daily basis to keep the cell density within the aforementioned range. When the maximum working volume per flask was reached, additional flasks were used.

### 4.5. Gas-Permeable Flask Culture

Cells were cultured at a density of 1.2–2 × 10^6^ cells/mL into gas-permeable flasks (Innovel, Suzhou, China). Fresh medium was supplemented on a daily basis to keep the cell density within the aforementioned range. When the maximum working volume per flask was reached, additional flasks were used.

### 4.6. Wave Bioreactor Culture

Cells were seeded into a 2 L cell bag (Sinobiocan, Shanghai, China) with a working volume of 1000 mL on day 2 of culture. Prior to inoculation, the culture medium was pre-equilibrated at 37 °C with 5% CO_2_ for at least 24 h. The culture was initiated with 1–6 × 10^8^ cells at a density of 0.3–1.5 × 10^6^ cells/mL in T cell culture medium. Cells were subsequently diluted daily to maintain the seeding density until the maximum volume of 1000 mL was reached. One day after reaching the maximum volume, perfusion was initiated. The perfusion volume, rocking angle, and speed were dynamically adjusted according to the cell growth status.

### 4.7. Surface Marker Detection by Flow Cytometry

The CAR and surface protein expression levels were evaluated with a CytoFLEX S flow cytometer (Beckman Coulter, Inc., Brea, CA, USA). For surface staining, no less than 5 × 10^5^ cells were aliquoted into 5 mL flow cytometry tubes, followed by two washes with 1 mL of wash buffer (phosphate-buffered saline (PBS) supplemented with 2% fetal bovine serum (FBS)); each wash step was performed via centrifugation at 300× *g* for 5 min, and supernatants were discarded after centrifugation. Subsequently, cells were incubated with fluorophore-conjugated antibodies targeting the specific surface markers of interest, with the incubation carried out at 2–8 °C for 30 min. After the completion of staining, cells were subjected to another wash and centrifugation step and then resuspended in 100 µL of wash buffer. For cell viability assessment, 2 µL of 7-AAD Viability Staining Solution was mixed with the cells and they were incubated at room temperature for 10 min before proceeding with flow-cytometric analysis.

For apoptosis analysis, cells were first subjected to two washes with flow cytometry wash buffer and then resuspended in 100 µL 1× binding buffer; then, 5 µL PE Annexin V was added and 5 µL of 7-AAD was incorporated into each sample. This was followed by 10 min incubation at room temperature under dark conditions to avoid fluorophore quenching. After the staining process was completed, each sample received 400 µL of 1× binding buffer, and the prepared samples were immediately analyzed using flow cytometry.

The antibodies used are listed in Supplementary Table S1.

### 4.8. Intracellular Cytokine Detection by Flow Cytometry

The 1:1 E:T co-incubation of CAR-T and A673 cells was performed for 24 h, with the culture maintained in a humidified incubator at 37 °C and 5% CO_2_. Brefeldin A (BioLegend, San Diego, CA, USA) was administered in the final 4–6 h of the stimulation period to inhibit cytokine secretion. After completion of the co-culture, cells were harvested and subjected to two washes with flow cytometry wash buffer, followed by staining with BD Horizon™ Fixable Viability Stain 510 (BD Pharmingen, San Diego, CA, USA) and MonoRab™ Rabbit Anti-scFv Cocktail [iFluor 488] (GenScript, Nanjing, China). Subsequent to surface staining, cells were fixed and permeabilized using the Cyto-Fast™ Fix/Perm Buffer Set (BioLegend, San Diego, CA, USA), strictly following the protocol provided by the manufacturer. Thereafter, intracellular staining was conducted using antibodies specific to interferon-γ (IFN-γ), tumor necrosis factor-α (TNF-α), and granzyme B (GZMB). A complete list of the antibodies used is provided in Supplementary Table S1.

### 4.9. Nuclear Transcription Factor TOX Detection by Flow Cytometry

The 1:1 E:T co-incubation of CAR-T and A673 cells was performed for 24 h, with the culture maintained in a humidified 37 °C incubator containing 5% CO_2_. At the end of the co-culture period, cells were collected and underwent two washes with flow cytometry wash buffer. Following washing, cells were stained with BD Horizon™ Fixable Viability Stain 510 (BD Pharmingen, San Diego, CA, USA) and MonoRab™ Rabbit Anti-scFv Cocktail [iFluor 488] (GenScript, Nanjing, China) for surface marker labeling. After surface staining was finished, cells were fixed and permeabilized using the True-Nuclear™ Transcription Factor Buffer Set (BioLegend, San Diego, CA, USA), adhering strictly to the manufacturer’s recommended protocol. Subsequently, the cells were stained with anti-TOX antibodies to detect TOX expression. All antibodies utilized in this experiment are listed in Supplementary Table S1.

### 4.10. CD107a Expression Detection by Flow Cytometry

The 1:1 E:T co-incubation of CAR-T and A673 cells was performed for 24 h, with the culture maintained at 37 °C and 5% CO_2_; anti-CD107a antibody was added to the culture system during this period. Following incubation, cells were harvested and subjected to two washes and then stained with MonoRab™ Rabbit Anti-scFv Cocktail [iFluor 488] (GenScript, Nanjing, China) at 2–8 °C for 30 min. After an additional wash step, cells were resuspended in 100 µL of flow buffer, followed by the addition of 2 µL 7-AAD Viability Staining Solution. Samples were incubated at room temperature for 10 min prior to flow-cytometric analysis. All antibodies employed in this experiment are listed in Supplementary Table S1.

### 4.11. Calcein AM-Based Cytotoxicity Assay

The A673 cell line was stained with 5 µM Calcein-AM (Aladdin, Shanghai, China) and incubated at 37 °C for 30 min in a non-CO_2_ incubator. After the staining process, the labeled target cells were co-incubated together with CAR-T cells in 96-well plates at three different E:T ratios (25:1, 5:1, and 1:1) for 2.5 h, with the culture maintained in a humidified incubator. Following the co-incubation period, supernatants were harvested, and the fluorescence intensity (FI) of each sample was measured using a microplate reader (PerkinElmer Victor X3, PerkinElmer, Inc., Shelton, CT, USA). The excitation and emission wavelengths for FI detection were set to 485 nm and 530 nm, respectively. The corrected percent cytotoxicity was calculated using the following formula: corrected % lysis = (FI from cytotoxic T lymphocyte assay − FI from spontaneous release)/(FI from maximum release − FI from spontaneous release).

### 4.12. Luciferase-Based Cytotoxicity Assay

Target cells expressing luciferase were plated into 96-well plates at 1 × 10^4^ cells per well. CAR-T cells were subsequently introduced at an E:T ratio of 0.5:1, with the final volume per well adjusted to 200 µL. After thorough mixing via gentle pipetting, the plates were incubated at 37 °C in a humidified incubator containing 5% CO_2_ for 24 h. Following the incubation period, 2 µL of D-luciferin solution was introduced into each well; the contents were gently mixed using a pipette, and the plates were incubated for an additional 15 min at 37 °C. The luminescence intensity of each well was then measured using a microplate reader. Cytotoxicity was calculated with the following formula: cytotoxicity (%) = 1 − [(luminescence of experimental group − luminescence of positive control)/(luminescence of negative control − luminescence of positive control)] × 100%.

### 4.13. Lipid Droplet Analysis

Intracellular lipid droplets were detected using the green fluorescent probe BODIPY 493/503 (Beyotime, Shanghai, China). According to the manufacturer’s instructions, BODIPY 493/503 (1000×) and Hoechst 33,342 (100×) were diluted in assay buffer to prepare the staining solution. A total of 1 × 10^6^ cells were collected and centrifuged at 700× *g* for 5 min to remove the supernatant. The cell pellet was resuspended in 500 μL of staining solution and incubated for 30 min at room temperature in the dark. Cells were then centrifuged again at 700× *g* for 5 min, the supernatant was discarded, and the pellet was resuspended in 100 μL of PBS. Finally, 2 μL of 7-AAD was added to exclude dead cells, and samples were analyzed by flow cytometry.

### 4.14. Mitochondrial Status Assessment

The mitochondrial mass was evaluated using MitoTracker™ Green FM (Yeasen, Shanghai, China). A 100 nM staining solution was prepared according to the manufacturer’s instructions. A total of 1 × 10^6^ cells were collected and centrifuged at 700× *g* for 5 min to remove the supernatant. The cell pellet was resuspended in 500 μL of staining solution and incubated for 30 min under standard cell culture conditions (37 °C, 5% CO_2_) in the dark. Cells were then centrifuged again at 700× *g* for 5 min, the supernatant was discarded, and the pellet was resuspended in 100 μL of PBS. Finally, 2 μL of 7-AAD was added to exclude dead cells, and samples were analyzed by flow cytometry.

The mitochondrial membrane potential was assessed using tetramethylrhodamine ethyl ester (TMRE; Beyotime, Shanghai, China). The TMRE staining solution was prepared by diluting the 1000× stock solution in assay buffer according to the manufacturer’s instructions. A total of 1 × 10^6^ cells were collected and centrifuged at 700× *g* for 5 min to remove the supernatant. The cell pellet was resuspended in 500 μL of staining solution and incubated for 30 min under standard cell culture conditions (37 °C, 5% CO_2_) in the dark. Cells were then centrifuged again at 700× *g* for 5 min, the supernatant was discarded, and the pellet was resuspended in 100 μL of PBS. Finally, 2 μL of 7-AAD was added to exclude dead cells, and samples were analyzed by flow cytometry. Cells treated with carbonyl cyanide m-chlorophenyl hydrazone (CCCP) for 20 min were used as a negative control.

### 4.15. Repeated Antigen Stimulation Assay

CAR-T cells were expanded using distinct manufacturing systems for 14 days. For subsequent antigen stimulation, tissue culture flasks were pre-coated with in-house-produced CD99 protein. The amount of protein used for coating was determined based on the incubation volume, seeking to achieve a final working concentration of 500 ng/mL during stimulation. The flasks were incubated at 37 °C for 4 h prior to cell seeding. Cells were stimulated at 24 h intervals without the addition of exogenous cytokines. A total of ten antigen stimulation rounds were performed, and CAR-T cells were collected 24 h after the final stimulation for downstream analyses.

### 4.16. Bulk RNA-seq

Total RNA was extracted from CAR-T cells using TRIzol (ThermoFisher, Waltham, MA, USA), according to the manufacturer’s instructions. Sequencing was performed by the Beijing Genomics Institute (BGI), Shenzhen. Raw sequencing data were filtered using SOAPnuke [50] and aligned to the human reference genome GRCh38 using HISAT2 (v2.2.1) [51]. Gene expression was quantified with RSEM (v1.3.1) [52]. Differential expression analysis was performed using DESeq2 (v1.34.0) [53] with a paired donor design, considering genes with |log2FC| ≥ 0.58 and adjusted *p* < 0.05 as significant DEGs. Gene set enrichment analysis (GSEA) was conducted using clusterProfiler (v4.4.4) [54] to identify significantly altered pathways (FDR < 0.05). Principal component analysis (PCA) [55] was performed on variance-stabilized transformed (VST) data after removing zero-variance genes using the R function prcomp. Gene set variation analysis (GSVA, v1.44.5) [56] was applied to estimate pathway activity across samples.

### 4.17. Targeted Metabolomics

Frozen CAR-T cell samples were quickly transferred from −80 °C to ice and extracted with 200 µL of pre-cooled extraction solvent (methanol–acetonitrile–water = 2:2:1, −20 °C) containing 2 µL of isotopic internal standard mixture. Samples were sonicated on ice for 5 min at 4 °C, frozen at −80 °C for 30 min, and centrifuged at 12,000× *g*, 4 °C for 15 min. The supernatant was collected twice and transferred to injection vials for liquid chromatography–quadrupole time-of-flight mass spectrometry (LC/Q-TOF) analysis. Chromatographic separation was performed on a Waters BEH Amide column (2.1 × 100 mm, 1.7 µm) using mobile phase A (15 mM ammonium acetate in water with 0.3% ammonia) and mobile phase B (15 mM ammonium acetate in 90% acetonitrile with 0.3% ammonia), with an injection volume of 2 µL and a column temperature of 40 °C. Mass spectrometry was conducted on an Agilent 6545 Q-TOF in positive and negative electrospray ionization (ESI) modes, with the following source parameters: gas temperature 300 °C, sheath gas temperature 325 °C, drying gas flow 8 L/min, sheath gas flow 11 L/min, nebulizer pressure 40 psi, capillary voltage 3500 V, and nozzle voltage 500 V for positive mode and 1000 V for negative mode. Target metabolites were identified using an in-house standard database based on the accurate mass, MS/MS fragments, and retention time, and relative quantification was performed using internal standards. Values below the quantification limit were set to 0. Differential metabolites between groups were determined by paired *t*-tests with *p* < 0.05 and subsequently subjected to KEGG pathway enrichment analysis.

### 4.18. Mouse Models

A total of 15 female NOD.Cg-Prkdc^scid^ Il2rg^tm1^/Vst (NPG) mice (8 weeks old, 20–22 g) were purchased from Beijing Vitalstar Biotechnology Co., Ltd. (Beijing, China) and housed at the Laboratory Animal Center of Wuhan University of Science and Technology. Mice were maintained in a sterile environment under a 12 h light/dark cycle at approximately 23 °C and 50% relative humidity, with ad libitum access to food and water. All animal experiments were approved by the Animal Ethics Committee of Wuhan University of Science and Technology (approval no. WKD-Shi-01; date: 14 March 2025). Each mouse was subcutaneously injected with 1.5 × 10^6^ A673 cells suspended in 100 μL of saline (0.9% NaCl). Tumor progression was monitored by measuring the tumor volume. On day 5 post-injection, mice were randomly assigned to three groups based on their initial tumor volumes (n = 5 per group) and received intravenous injections of 1.2 × 10^7^ CD99 CAR-T cells cultured in either gas-permeable flasks or the wave bioreactor or 100 μL of saline (0.9% NaCl) alone. Tumor growth was measured every two days. Blinding was applied during tumor measurements to minimize observer bias. Mice were monitored daily for health and behavior and were euthanized on day 17—or earlier if humane endpoints were reached (e.g., >20% body weight loss, persistent hunched posture, severe weakness, inability to eat or drink, labored breathing, or excessive tumor burden). Tumor tissues were subsequently collected for flow-cytometric analysis.

### 4.19. Statistical Analysis

No statistical methods were applied to pre-define sample sizes; instead, our sample sizes were either consistent with those reported in prior publications or determined based on our own experimental experience. Biological replicates correspond to independent experiments conducted using T cells isolated from at least three different healthy donors. Specific details regarding the number of experimental replicates and the types of replicates (technical or biological) are provided in the respective figure legends. Statistical analyses were performed using the Prism 9.0 software (GraphPad Software). The specific analytical methods employed and the definition of statistical significance are indicated in the corresponding figures and their associated legends. The Shapiro–Wilk test was used to assess the normality of the data. For group comparisons, an unpaired Student’s *t*-test or analysis of variance (ANOVA) with Šídák’s post hoc multiple comparisons test was utilized, depending on the experimental design. A *p*-value < 0.05 was considered statistically significant. All data are presented as the mean ± standard error of the mean (SEM). No data points were excluded from the analysis.

## 5. Conclusions

The dynamic cultivation of CD99 CAR-T cells induces mitochondrial-centered metabolic reprogramming, characterized by enhanced TCA cycle activity and sustained oxidative phosphorylation. This metabolic state supports memory-like phenotypes, reduces exhaustion, and maintains cytotoxic function under repeated antigen stimulation, providing a mechanistic rationale for improvements in CAR-T cell persistence and antitumor efficacy in clinical applications.

## Data Availability

All data are contained within the article and Supplementary Materials. The raw metabolomics data have been deposited in the National Genomics Data Center (NGDC) OMIX database (accession number: OMIX014019). Further inquiries can be directed to the corresponding author.

## Supplementary Materials

The following supporting information can be downloaded at: https://www.mdpi.com/article/10.3390/ijms27020607/s1.

## Abbreviations

The following abbreviations are used in this manuscript:

CAR	Chimeric Antigen Receptor
MHC	Major Histocompatibility Complex
TN	Naïve T Cell
TSCM	Stem Cell-Like Memory T Cell
TCM	Central Memory T Cell
TEM	Effector Memory T Cell
TEFF	Effector T Cell
GMP	Good Manufacturing Practice
PCA	Principal Component Analysis
SPICE	Simplified Presentation of Incredibly Complex Evaluations
GSEA	Gene Set Enrichment Analysis
GSVA	Gene Set Variation Analysis
OXPHOS	Oxidative Phosphorylation
TCA	Tricarboxylic Acid Cycle
FAO	Fatty Acid Oxidation
ATP	Adenosine Triphosphate
NES	Normalized Enrichment Score
FDR	False Discovery Rate
DMEM	Dulbecco’s Modified Eagle Medium
FBS	Fetal Bovine Serum
PBS	Phosphate Buffer Saline
E:T	Effector-to-Target Ratio
RNA-seq	RNA Sequencing
TILs	Tumor-Infiltrating Lymphocytes
CCCP	Carbonyl Cyanide m-Chlorophenyl Hydrazone
MTG	MitoTracker Green
TMRE	Tetramethylrhodamine Ethyl Ester
FMN	Flavin Mononucleotide
NPG	NOD.Cg-Prkdc^scid^ Il2rg^tm1^/Vst
EF-1α	Elongation Factor 1 Alpha
MOI	Multiplicity of Infection
VST	Variance-Stabilizing Transformation
LC/Q-TOF	Liquid Chromatography–Quadrupole Time-of-flight Mass Spectrometry
ANOVA	Analysis of Variance
SEM	Standard Error of the Mean
KEGG	Kyoto Encyclopedia of Genes and Genomes
CDX	Cell line–derived Xenograft
NAD+	Nicotinamide Adenine Dinucleotide (oxidized form)
NADH	Nicotinamide Adenine Dinucleotide (reduced form)
AMP	Adenosine Monophosphate
ADP	Adenosine Diphosphate
