# Supplementary material for: Upregulation of the TCA Cycle and Oxidative Phosphorylation Enhances the Fitness of CD99 CAR-T Cells Under Dynamic Cultivation
Source: Int J Mol Sci. 2026 Jan 7;27(2):607. doi: 10.3390/ijms27020607 (PMC12841239; doi:10.3390/ijms27020607)
Supplement: Supplementary file 1 [file ijms-27-00607-s001.zip › Supplementary figure legend.pdf]

Figure S1 Parameter Optimization of Wave Bioreactor

A Culture conditions comparing two sets of rocking parameters over the culture period. **B** Fold expansion of total T cells transduced with CD99 CAR under different wave bioreactor parameters over 14 days ($n = 2$ biologically independent replicates). C Viability of total T cells transduced with CD99 CAR under different wave bioreactor parameters over 14 days ($n = 2$ biologically independent replicates). Data are presented as mean ± SEM. Statistical analysis was performed using two-way ANOVA followed by Šídák's multiple comparisons test. Statistical significance is indicated as follows: * $p < 0.05$, ** $p < 0.01$ and *** $p < 0.001$; ns, not significant.
