# Supplementary material for: Upregulation of the TCA Cycle and Oxidative Phosphorylation Enhances the Fitness of CD99 CAR-T Cells Under Dynamic Cultivation
Source: Int J Mol Sci. 2026 Jan 7;27(2):607. doi: 10.3390/ijms27020607 (PMC12841239; doi:10.3390/ijms27020607)
Supplement: Supplementary file 1 [file ijms-27-00607-s001.zip › Supplementary Table S1.pdf]

Supplementary Table S1: Antibodies Used for Flow Cytometry.

Marker	Fluorophore	Catalog #	Clone	Dilution	Application
SCFV	iFluor 488	Genscript A02286	/	1:50	FC
CD25	PE	Biolegend 302606	BC96	1:50	FC
CD69	BV421	Biolegend 310930	FN50	1:50	FC
CD71	BV650	Biolegend 334116	CY1G4	1:50	FC
CD45RA	BV421	Biolegend 304130	HI100	1:50	FC
CCR7	PE	Biolegend 353204	G043H7	1:50	FC
PD-1	PE	Biolegend 329906	EH12.2H7	1:50	FC
TIM-3	BV421	Biolegend 345008	F38-2E2	1:50	FC
TIM-3	PE-cy7	Biolegend 345013	F38-2E2	1:50	FC
LAG-3	BV650	Biolegend 369316	11C3C65	1:50	FC
Annexin V	PE	Biolegend 640908	/	1:25	FC
TOX	PE	Invitrogen 12-6502-82	TXRX10	1:50	FC
CD107a	PE	Biolegend 328608	H4A3	1:200	FC
GZMB	BV421	Biolegend 369414	QA16A02	1:50	FC
IFN-γ	PE-cy7	Biolegend 502528	4S.B3	1:50	FC
TNF-α	BV650	Biolegend 502938	Mab11	1:50	FC
