# Supplementary figures and images for: Upregulation of the TCA Cycle and Oxidative Phosphorylation Enhances the Fitness of CD99 CAR-T Cells Under Dynamic Cultivation
Source: Int J Mol Sci. 2026 Jan 7;27(2):607. doi: 10.3390/ijms27020607 (PMC12841239; doi:10.3390/ijms27020607)

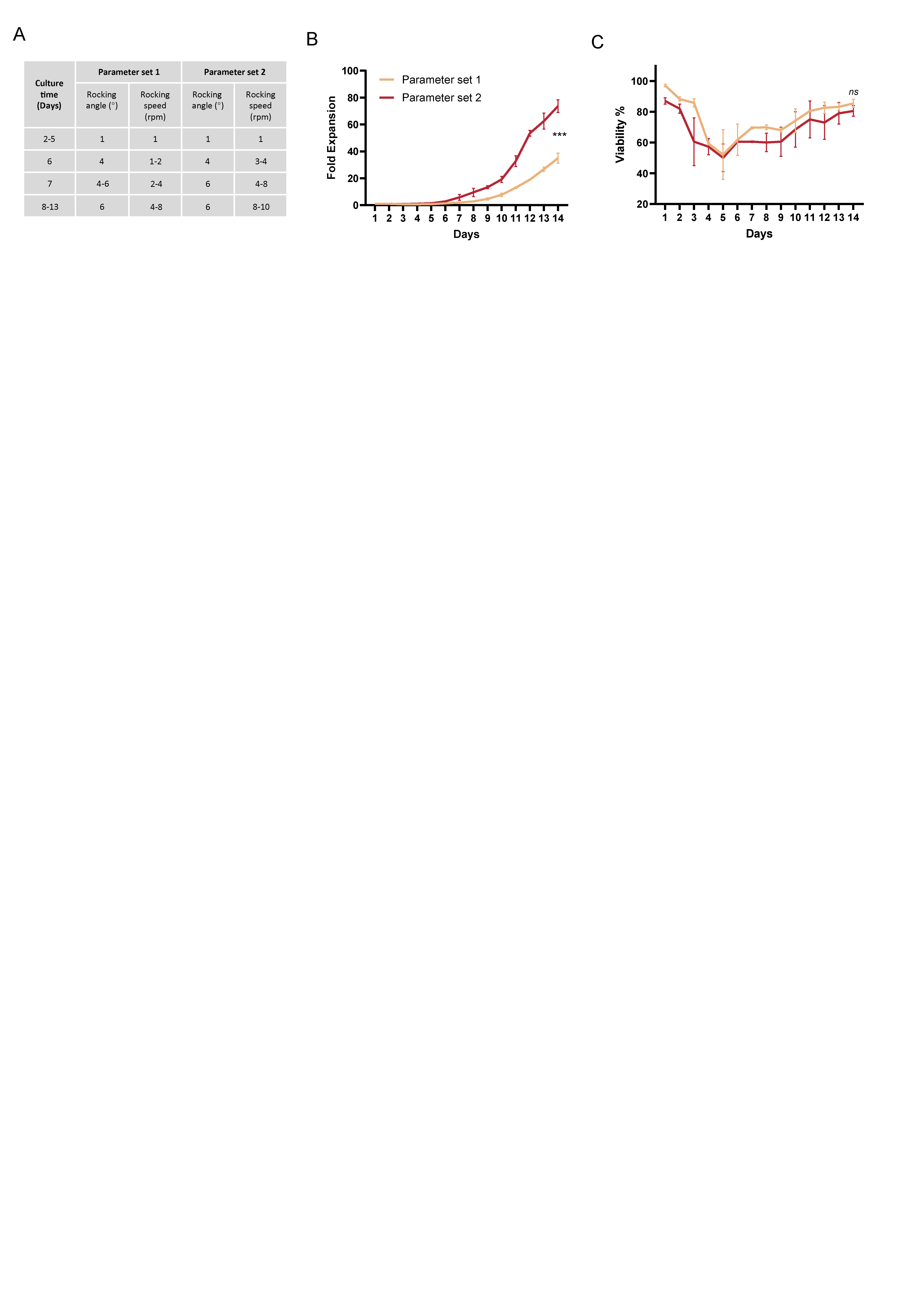

Supplement: Supplementary file 1 [file ijms-27-00607-s001.zip › FigS1.tif]
